# Knowledge, Perception, and Willingness towards Immunization among Bangladeshi Population during COVID-19 Vaccine Rolling Period

**DOI:** 10.3390/vaccines9121449

**Published:** 2021-12-07

**Authors:** Md. Sazzadul Bari, Md. Jamal Hossain, Foyez Ahmmed, Md. Moklesur Rahman Sarker, Labony Khandokar, Aperajita Paul Chaithy, Farina Aziz, Saikat Mitra, Talha Bin Emran, Md. Saiful Islam, Md. Rabiul Islam, Isa Naina Mohamed

**Affiliations:** 1 Department of Pharmacy, State University of Bangladesh, 77 Satmasjid Road, Dhanmondi, Dhaka 1205, Bangladesh; sazzadulbari1516@gmail.com (M.S.B.); moklesur2002@yahoo.com (M.M.R.S.); chaitypaul009@gmail.com (A.P.C.); 2 Department of Statistics, Comilla University, Kotbari, Cumilla 3506, Bangladesh; foyez.sbi@gmail.com; 3 Department of Pharmacy, East West University, A/2 Jahurul Islam Avenue, Dhaka 1212, Bangladesh; labony.k123@gmail.com; 4 Department of Pharmacy, University of Asia Pacific, 74/A Green Road, Farmgate, Dhaka 1215, Bangladesh; farina@uap-bd.edu (F.A.); robi.ayaan@gmail.com (M.R.I.); 5 Department of Pharmacy, Faculty of Pharmacy, University of Dhaka, Dhaka 1000, Bangladesh; saikatmitradu@gmail.com; 6 Department of Pharmacy, BGC Trust University Bangladesh, Chittagong 4381, Bangladesh; talhabmb@bgctub.ac.bd; 7 Department of Public Health and Informatics, Jahangirnagar University, Savar, Dhaka 1342, Bangladesh; islam.msaiful@outlook.com; 8 Pharmacology Department, Medical Faculty, Universiti Kebangsaan Malaysia (The National University of Malaysia), Cheras, Kuala Lumpur 56000, Malaysia

**Keywords:** COVID-19, vaccine willingness, mean knowledge score, mean perception score, Bangladesh

## Abstract

Vaccine willingness among the mass populace, as well as their proper knowledge and perception regarding vaccines and the vaccination process, may contribute extensively towards attaining their anticipated vaccination rates. The current study endeavored to ascertain the Bangladeshi population’s knowledge, perception, and willingness towards COVID-19 vaccination. Relevant information was collected from 1201 adults aged 18 years or older by employing an online-based survey from 1 to 30 July 2021. Descriptive statistics, the chi-square (χ^2^) test, and a binary logistic regression analysis were applied in order to compare the extent of knowledge and perception prevalent among different demographic groups and correlate such prevalence with respective vaccine willingness. The participants expressed mean (± standard deviation) knowledge and perception scores of 6.48 ± 1.13 out of 8 and 5.37 ± 1.22 out of 7, respectively. A multivariate analysis confirmed the significant association (*p* < 0.05) of gender, age, and family income with the knowledge score, whereas age and knowledge level significantly influenced perception. Current living area, family income, and age were considerable contributors to COVID-19 vaccine willingness. Overall vaccine willingness was found to be significantly curtailed by inadequate knowledge (AOR 0.514, CI 95% 0.401–0.658, *p* < 0.001) and perception (AOR 0.710, CI 95% 0.548–0.920, *p* = 0.010) among the participants. All of the concerned authorities’ efforts are warranted in order to improve public understanding, perception, and inclination towards vaccination.

## 1. Introduction

Defined as the “Severe Acute Respiratory Syndrome Coronavirus 2” (SARS-CoV-2), the seventeenth member of the coronavirus family marked its entry into human history by December 2019 as reports of a highly contagious version of pneumonia started emerging from Wuhan, Hubei province, China [1]. Eventually, the World Health Organization (WHO) designated the term “Coronavirus Disease-2019” or “COVID-19” in short, to address the disease globally and declared it a pandemic on 11 March 2020 [2]. As of May 2021, the pandemic has already entered its second wave of infection, affecting over 172 million people in at least 223 countries and has claimed the lives of over 3.6 million people worldwide [3]. The exceptional infectivity of SARS-CoV-2 may be attributed to the capacity of an infected person to become contagious even before presenting clinical symptoms. Clinical modeling has estimated that a patient starts transmitting the virus around 2.5 days earlier than the exhibition of discernible symptoms [4]. This, in turn, makes it very difficult to identify and isolate potential sources of contamination from within the mass populace. Therefore, the only genuine hope for humankind lies in the successful development of effective vaccines, as it would lead to the development of mass immunity against this pathogen. Outstanding efforts from both government- and private-funded research organizations have culminated in the development of around 91 vaccine candidates, which are currently undergoing phase-III and phase-IV clinical trials for further evaluation of their efficacy and safety [5]. Moreover, the global urgency to withstand the aggression of the COVID-19 pandemic has already led to the conditional approval of certain vaccines in many countries, including ChAdOx1 (also known as AZD1222) from the collaboration of University of Oxford-AstraZeneca (Sweden-UK), Sputnik V from Gamaleya Research Institute (Russia), BNT162b2 from the collaboration of Pfizer-BioNTech (USA-Germany) and mRNA-1273 from the government-sponsored efforts of Moderna (USA) [6].

Bangladesh, a South-Asian country of around 165 million people, recorded its first COVID-19 case from an expatriate on 8 March 2020, while the first death was reported on 19 March 2020 [7]. Like many countries, Bangladesh is currently experiencing the second wave of the pandemic, which has proved to be more devastating compared to the first one, as daily numbers of both confirmed cases and deaths reached record figures of 7626 and 112 on 8 April 2021 and 20 April 2021, respectively. The total number of confirmed cases as of early June 2021 has reached over 800,000, whereas the cumulative death count has risen above 12,000 [8]. However, the country was prompt in its response to adopting a nationwide vaccination policy as the ChAdOx1 vaccine of Oxford-AstraZeneca was approved in early January 2021, and arrangements were secured with Serum Institute of India to import the vaccine under the brand name of “Covishield” [9]. The administration of the ChAdOx1 vaccine, first at a low dose and then at the standard dose, was reported to attain an efficacy of 90.0% [10]. As of the end of May 2021, Bangladesh has successfully administered both doses of the vaccine to a little over 4 million people, while the first dose has been administered to more than 5.8 million people [11].

Swift and extensive vaccination of the mass populace is contingent upon high vaccine acceptance rates among the common people. Irrespective of the disease, a general unwillingness towards vaccination has been institutionally recognized by the WHO Strategic Advisory Group of Experts on Immunization, which has been termed as “Vaccine hesitancy” and recognized as a major threat to global healthcare management. Moreover, the exceptionally accelerated development of COVID-19 vaccines and the subsequent lack of extensive safety profiling, along with the mass circulation of misinformation, may have further aggravated the high skepticism among the general population towards the COVID-19 vaccine. Eventually, a global survey covering 19 countries delineated that vaccine acceptance varied largely from 55% (Russia) to 90% (China) between different nations [12]. However, an earlier study of the Chinese population demonstrated that only 28.7% of their respondents were definitely positive, whereas 54.8% answered “Probably yes” when questioned about getting vaccinated, indicating a non-linear association between willingness and actual intention for vaccination [13]. Other similar studies focusing on the willingness of the respective populace of individual countries to get the COVID-19 vaccine have illustrated a 57.7% willingness in Greece [14] and a 64.72% willingness in Saudi Arabia [15]. Moreover, an Indonesian study enumerated that based on the extent of efficacy and safety of the vaccines, vaccine acceptance could vary significantly, ranging from 67.0% (efficacy 50%) to 93.3% (efficacy 95%) [16].

A systematic review covering more than a thousand studies from all around the globe has postulated three major sources of influences which diminish the willingness of the general populace for vaccination viz. contextual (demographic) influences, vaccine/vaccination knowledge-based influences and individual/social group (perceptive) influences [17]. Accordingly, different studies around the world (including in Bangladesh) have already endeavored to evaluate the influence of knowledge and perception regarding COVID-19 vaccination on the general willingness to get vaccinated [18,19,20,21,22,23]. In this cross-sectional study, we have endeavored to ascertain the current level of knowledge and perception that is prevalent among different socio-economic groups of the Bangladeshi population as vaccination against COVID-19 has already begun in the country. Subsequently, we have attempted to use vigorous statistical analysis to correlate this information with the overall willingness of our sample populace to receive vaccination. Although previous efforts were put forth to assess vaccine willingness in the same population [18], they did not take into consideration the actual extent of public knowledge and perception, which have been identified to significantly affect vaccine acceptance [17]. Furthermore, while another endeavor did ascertain the knowledge and attitude regarding vaccine willingness [20], they did so while vaccination was still far from actually happening both in Bangladesh and any of its neighboring countries and the public concept was still tenuous, strained and apprehensive. The current work, however, was undertaken while vaccination was already enforced in many parts of the world, including the Indian sub-continent, and reports of its efficacy as well as possible adverse effects had also started emerging. It is our postulation that these reports can impose significant impacts on the public perception of vaccination, which in turn may alter the mass acceptance of vaccines. Thus, our study is focused on the association of vaccine willingness with public knowledge and perception while vaccination is already underway and reports on their efficacy, abundance and possible side-effects are also being communicated to the mass populace.

## 2. Materials and Methods

### 2.1. Study Design

This nationwide study was carried out in the form of a web-based cross-sectional survey. Bangladeshi people of 18 years of age or older from both rural and urban areas who had minimum access to the internet were targeted for sampling and data collection. The questionnaire contained 21 questions and was initially compiled in the English version, then translated into the Bangla version to be better understood by all classes of people. A native speaker of Bangla who had graduated from a medical institution and was professionally fluent in English verified the forward and backward translation of the questionnaire. The current research contained four sections categorized as section A: socio-demographic variables, section B: knowledge-based eight questions, section C: perception-based questions, and section D: willingness to be vaccinated against COVID-19 related query. The survey was initiated with several socio-demographic questions such as age, gender, level of schooling, family monthly income level, including the current living area of the respondents.

### 2.2. Participants and Data Collection

Members of the Bangladeshi population aged 18 years or above who had frequent access to the internet and were familiar with google survey responses were primarily the potential samples of the study. Prolonged confinements and social restrictions have compelled people into being more active on social media in order to keep up with current events and maintain social interactions. As a result, social media was approved as an effective method of communication. After creating the questionnaire, the google link of the designed research was circulated among the well-known social media platforms including Facebook groups and pages, Messenger groups, WhatsApp groups, and several other social networks in order to collect nationwide data. Moreover, to ensure proper diversity and rational variability in the responses, the individual social platforms were selected carefully in order to cover the general population of all ages and classes rather than particular sections. Both Bangla and English versions of the questionnaire were shared in order to facilitate the comprehension of the people responding to the survey. The Bangladeshi ethnic people who lived in Bangladesh during the study and understood the objectives and questions of the study participated in this survey. Exclusion criteria included medically sick, addicted, and acutely mentally ill people. In addition, no participants were paid for their participation in this survey.

Moreover, all of the collected raw data were confidentially maintained and in an excel sheet. Before beginning to answer the questionnaire, all of the respondents closely looked at the brief description of the study, which contained the aim, anonymity announcement, protocol, and instructions for responding to the survey. The study also maintained all of the privacy parameters and avoided any irrelevant questions in the questionnaire. Electronic consent was mandatorily collected from all of the respondents before participation in the survey. The current online-based data collection was done by following all of the protocols and guidelines proclaimed by the World Medical Declaration of Helsinki (2018). In addition, the ethical review committee of the State University of Bangladesh, Dhaka, Bangladesh, has critically reviewed and approved all of the ethical points of the current human perception-based study and provided an approval number (2021-04-13/SUB/ERC/0003, Date: 13 April 2021). The data collection timeframe of this online questionnaire-based study was from 1 to 30 July 2021.

### 2.3. Data Processing and Analysis

We collected 1201 responses within the above-mentioned data collection period, where 4.74% (*n* = 57) of data had to be excluded because of incompleteness. Finally, we analyzed 1144 responses after scrutinizing and eliminating the partial responses from the excel sheet. Three statistical methods, i.e., univariate, bivariate, and multivariate analyses, have been run to find and investigate the research outcomes. Descriptive statistics were applied to enumerate the frequency distributions and percentages of various socio-demographic factors in the study. In addition, the chi-square (χ^2^) test was conducted to find the potential association of covariates with the knowledge score, attitudes score, and willingness to get the COVID-19 vaccine. Finally, a logistic regression analysis was conducted to determine the association of various variables with the knowledge score, perception score, and willingness to be vaccinated after adjusting other factors. The marginal error or two-sided statistically significant level was less than 5% (*p* < 0.05) in the study for all of the statistical methods [24].

### 2.4. Assessment of KPW

In order to assess the knowledge and perception level towards the COVID-19 vaccination among the Bangladeshi population, we set eight knowledge-based questions (designated as K for Knowledge, K1–K8: “Yes = 1” vs. “No = 0,” score range: 0 to 8) and seven perception-based questions (designated as P for perception, P1–P7: “Yes = 1” vs. “No = 0,” score range: 0 to 7). In addition, to verify the willingness of the respondents to be vaccinated against COVID-19, a single question (designated as W for Willingness) was asked (Supplementary Material Table S1). After the initial drafting of the questionnaire, all of the queries were validated by the WHO recommended guidelines [25] and previously published pieces of literature [17,26,27] in the Bangladesh context. The participants’ scores were defined into good knowledge and perception (if the participants’ score was greater or equal to the average value) and poor knowledge and perception (if the participants’ score was less than the average value) [28].

## 3. Results

### 3.1. Demographic Characteristics

Among the 1144 participants who completely responded to this questionnaire-based survey, male and female were almost at a 1:1 ratio (*n* = 585; 51.1% and *n* = 559; 48.9%, respectively), illustrating a close balance of responses between both genders. Our respondents were categorized into three age groups, viz. 18–24 years, 25–40 years, and older than 40 years of age. A significant number of 797 respondents (69.7%) were from 18–24 years. In addition, two-thirds of the respondents (*n* = 756; 66.1%) have completed their Bachelor’s degree. A majority of the respondents (*n* = 855; 74.7%) participated in this online-based survey from urban areas, whereas more than one-fourth of the participants (*n* = 289; 25.3%) lived in rural areas.

The economic status of our respondents was well distributed across different social classes. Around one-thirds of the participants (*n* = 379; 33.1%) had a monthly family earning of BDT 25,000 (equivalent to around $300) or less. Furthermore, monthly incomes in the range of BDT 25,000–50,000 ($300–600) were had by the families of 47.6% respondents (*n* = 545) while a higher figure (greater than BDT 50,000) was had by only 19.2% respondents’ families (*n* = 220). Similarly, all of the socio-economic demographic variables with their descriptive statistics (distribution and percentage) are summarized in Table 1.

### 3.2. Knowledge, Perception, and Willingness (KPW)

**Figure 1:**
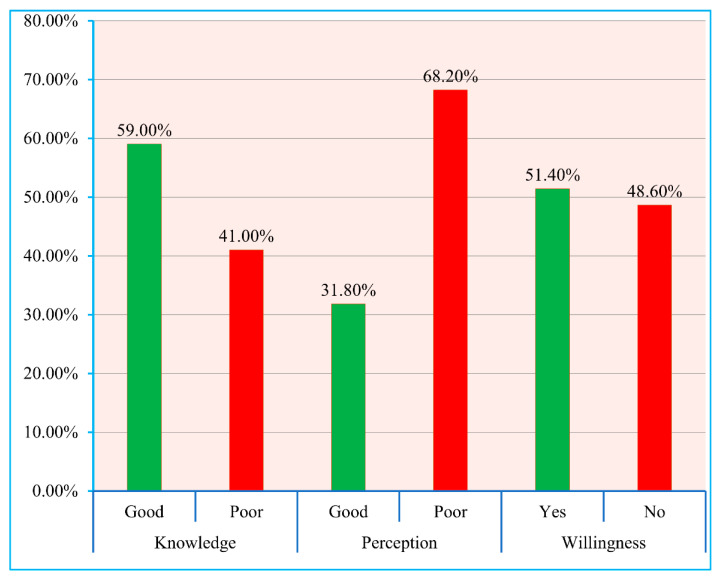
Percentage distributions of knowledge (good and poor), perception (good and poor), and willingness (yes and no) for taking COVID-19 vaccine among Bangladeshi population. All respondents’ scores were calculated through descriptive statistics and categorized into good knowledge and perception (if the participants’ score was greater than or equal to the mean value) and poor knowledge and perception (if the participants’ score was less than the mean value) [28].

The respondents’ knowledge level was evaluated based on eight queries and the average knowledge score was calculated at 6.48 ± 1.13 (mean ± standard deviation, SD) out of 8. Similarly, the perception of the participants were graded based on seven questions and the mean perception of the samples were measured at 5.37 ± 1.22. Accordingly, 675 respondents (59.0%) were characterized with a good level of knowledge whereas only 364 respondents (31.8%) exhibited good perception towards COVID-19 vaccine. Moreover, when asked about vaccination, slightly over half of the sample population (*n* = 588, 51.4%) expressed their willingness for receiving the vaccine (Figure 1). The mean knowledge scores of males and females were found to be 6.62 ± 1.12 and 6.33 ± 1.12, respectively. Almost similar perception scores (mean ± SD) for males and female were noted to be 5.09 ± 0.96 and 5.05 ± 0.99, respectively (Table 1).

Participants of the three age groups, viz. 18–24 years, 25–40 years and older than 40 years of age, demonstrated gradually increasing average knowledge scores of 6.44 ± 1.07, 6.49 ± 1.27 and 6.65 ± 1.26, respectively. Although the smallest age group of people exerted the highest perception score (5.15 ± 1.00), the 25–40 years-old respondents had the lowest perception score (4.76 ± 0.98). The participants with a Master’s degree or higher educational levels exhibited average knowledge and perception scores of 6.71 ± 0.96 and 4.94 ± 0.85. Respondents with Bachelor’s degree demonstrated a lower mean knowledge score (6.45 ± 1.11), but a higher mean perception score (5.09 ± 0.98). The average perception score for the HSC group rose even higher (5.13 ± 1.01) while the average knowledge score (6.45 ± 1.24) remained almost close to the Bachelor’s degree group. A further decline in the mean knowledge score (6.29 ± 1.51) was observed in the participants who received a comparatively lower level of education (SSC or lower). Likewise, they also demonstrated the lowest value for mean perception score (4.94 ± 1.10) compared to the other educational groups. The descriptive statistical information of all the demographic groups regarding their respective knowledge and perception levels has been summarized in Table 1 for further correspondence.

**Figure 2:**
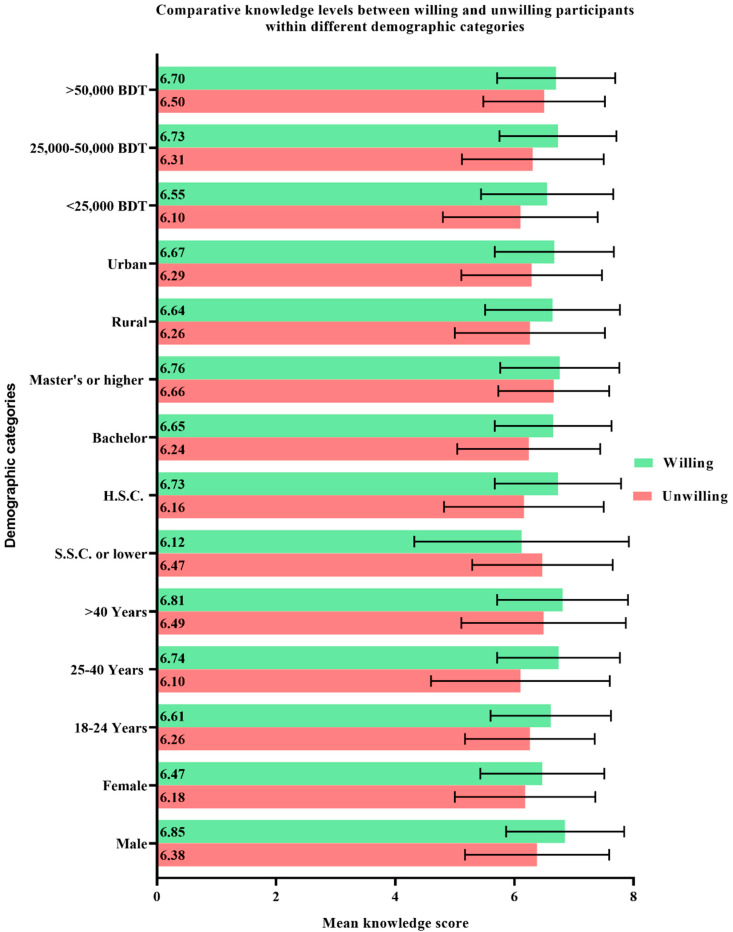
Comparative knowledge levels between willing and unwilling participants within different demographic categories expressed in terms of mean ± standard deviation.

**Figure 3:**
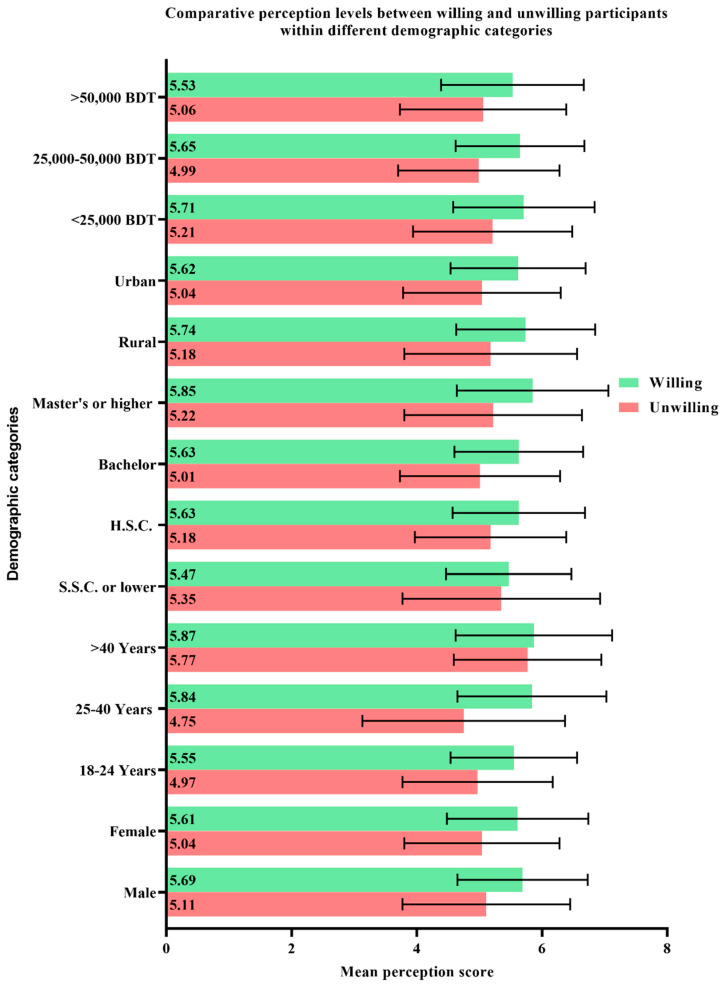
Comparative perception levels between willing and unwilling participants within different demographic categories expressed in terms of mean ± standard deviation.

When asked directly, only 51.4% respondents of the current study expressed their willingness to get vaccinated. Within every demographic category, the participants who expressed a willingness to receive vaccines expressed higher mean knowledge scores compared to the participants who were unwilling towards vaccination (Figure 2). On a similar note, the mean perception scores also varied significantly between the vaccine-willing and vaccine-unwilling participants within each demographic group (Figure 3).

### 3.3. Chi-Square (χ^2^) Analysis of KPW

The chi-square analysis revealed that several factors, such as gender, age, and education, were significantly (*p* < 0.05) associated with good knowledge scores, while age and the education were significantly (*p* < 0.1) correlated with a good perception towards COVID-19 vaccines in Bangladesh. In addition, the willingness to get a COVID-19 vaccine was influenced significantly (*p* < 0.1) by age, current living area, knowledge, and perception level toward the COVID-19 vaccine of the Bangladeshi population (Table 2).

A greater section of the male participants exhibited a good knowledge level compared to that of the female participants (*n* = 384; 65.6% vs. *n* = 291; 52.1%, *p <* 0.001). Respondents older than 40 years of age also exhibited a significantly (*p* < 0.001) greater degree (74.7%, *n* = 133) of good knowledge scores compared to those of the other two age groups viz. 18–24 and 25–40 years old (55.6%, *n* = 443 and 58.6%, *n* = 99, respectively). Furthermore, the prevalence of good knowledge was understandably most prominent in participants with the highest level of education (Master’s or higher). More than two-thirds of such respondents (69.8%, *n* = 97) were characterized by a good knowledge level, which was significantly (*p* < 0.05) higher than those of the other demographic groups with lesser levels of maximum education.

Over a third of the participants in the age group of 18–24 years (36.0%, *n* = 287) expressed a good perception towards COVID-19 vaccination. This was significantly higher (*p* < 0.001) compared to those of the other age groups of 25–40 years and older than 40 years as only 19.5% and 24.75 of their participants reached good perception scores, respectively. A comparatively lower extent (22.3%, *n* = 31) of good perception was also measured in participants with a Master’s degree or higher in their educational background. This was distinctly lower than the good perception levels of the other groups of participants with Bachelor’s (32.9%), higher secondary (34.0%) and secondary (32.4%) educational backgrounds.

Moreover, the people in the 25-to-40-years age group exhibited 9.5% and 11.5% higher willingness than the 18–24 years age group (59.8% vs. 50.3%, *p* = 0.055) and above-40-years age group (59.8% vs. 48.3%, *p* = 0.055), respectively. It is understandable that the urban people were more willing to get the COVID-19 vaccine than the rural people (53.0% vs. 46.7%, *p* = 0.065). In addition, a significantly higher willingness rate was observed during χ^2^-analysis for the participants with higher knowledge scores (57.6% vs. 42.4%, *p* < 0.001) and perception scores (56.9% vs. 48.8%, *p* = 0.001) than the poor-KP categorized people.

### 3.4. Logistic Regression Analysis of KPW

A logistic regression was applied to the multivariate analysis in order to investigate the several potential sociodemographic factors that might significantly correlate with knowledge, perception, and willingness to get COVID-19 vaccines among the Bangladeshi population. After adjusting other factors, the enumerated odds ratio, 95% confidence interval (CI), and the significant level (*p* values) of the association of various included factors are presented in Table 3.

The male participants were found to be 1.7 times more likely to be knowledgeable about COVID-19 vaccination in Bangladesh than the female respondents (95% CI = 1.367, 2.235; *p* < 0.001). Similarly, the knowledgeability about COVID-19 vaccination of the respondents older than 40 years of age was found to be significantly higher than that of the respondents of lower age groups viz. 18–24 years (18–24 vs. above 40: adjusted odds ratio, AOR = 0.467, 95% CI = 0.306, 0.714; *p* < 0.001) and 25–40 years (25–40 vs. above 40: AOR = 0.477, 95% CI = 0.298, 0.764; *p* = 0.002). Moreover, the respondents from families with lower income had around 33% lower knowledge scores than the participants from higher income families. (AOR = 0.665, 95% CI = 0.465, 0.951; *p* = 0.025).

With respect to the perception towards COVID-19 vaccines, the participants from the age range of 18–24 years demonstrated a near two-times higher positive perception compared to those older than 40 years (AOR = 1.944, 95% CI = 1.256–3.008, *p* = 0.003). In addition, more knowledgeable participants exerted 28% more positive perception than the less knowledgeable participants (AOR = 0.789, 95% CI = 0.606, 1.027, *p* = 0.078).

Table 3 also reveals that the respondents in the age group of 25–40 years expressed a significantly higher willingness to be vaccinated than those older than 40 years (AOR = 1.824, 95% CI = 1.170, 2.843; *p* = 0.008). In addition, rural participants showed around 28% lower willingness to be vaccinated than urban respondents (AOR = 0.719, 95% CI = 0.543, 0.951, *p* = 0.021). Contrarily, the participants from families with lower income (<25,000 BDT/month) exerted 1.4 times more willingness to be vaccinated than the participants from families with above 50,000 BDT/month income (AOR = 1.413, 95% CI = 0.993, 2.012, *p* = 0.055). Furthermore, the participants who achieved poor KP scores showed a significantly lower willingness to get the COVID-19 vaccine than their counterparts (vs. good knowledge, AOR = 0.514, 95% CI = 0.401, 0.658, *p* < 0.001, and vs. good perception, AOR = 0.710, 95% CI = 0.548, 0.920, *p* = 0.010). Likewise, Table 3 shows all of the variables influencing knowledge, perception, and willingness to get the COVID-19 vaccine among the Bangladeshi population.

## 4. Discussion

The fundamental assessment of KPW towards the COVID-19 vaccine among the Bangladeshi population was comparable to that of previous reports and replicated their findings with regard to a number of different variables, including gender, age, family income, and the current living area of the respondents [18,20]. However, the efforts of Abedin et al. were directed entirely towards the estimation of vaccine willingness and hesitancy while disregarding the measurement of the prevalence of both knowledge and perception within their sample populace [18]. Since both vaccine/vaccination-related knowledge and individual/social perception significantly influence vaccine acceptance [17,26], it is our understanding that the presentation of both knowledge-based and perceptive questions to the audience before seeking their opinion on vaccination may have affected their answers. Evidently, the current study presented a lower extent of vaccine willingness in Bangladesh (51.4%) compared to the 74.5% willingness that was reported by a previous study [18]. This idea was further reinforced as Islam et al. also evaluated the extent of vaccine-related knowledge and attitude towards COVID-19 vaccination among the Bangladeshi population and reported a lower margin of willingness (60.0%) within their respondents [20]. Similar measurements of vaccine acceptance were also reported among the Maltese (50%), Italian (53.7%), Russian (54.9%), Polish (56.3%), American (56.9%), Greek (57.7%), Nigerian (58.2%) and French (58.9%) populations [14,29,30]. On the contrary, the lowest rates of vaccine willingness were reported from Jordan (28.4%) and Kuwait (23.6%), whereas Ecuador (97.0%), Malaysia (94.3%), Indonesia (93.3%), and China (91.3%) exhibited the highest acceptance rates [12,16,31].

On account of the long-acting and devastating effects of the pandemic worldwide, including in Bangladesh, and vaccination being the only possible way to establish mass immunity, both of the percentages for knowledge (59.0%) and perception (31.8%) that were acquired in this study should be considered as unsatisfactory, in our opinion, when compared to previous reports [19,23,29]. A similar trend of insufficient knowledge levels was also reported by the earlier study of Islam et al. [20], indicating an overall insufficiency of adequate knowledge regarding the COVID-19 vaccine among the Bangladeshi population. As per our understanding, inexperience and ignorance regarding the implications of flu-like virus-borne epidemics like SARS (severe acute respiratory syndrome) and MERS (middle east respiratory syndrome) may have partially contributed to the overall reluctance of the Bangladeshi population towards COVID-19. Evidently, better knowledge regarding COVID-19 was demonstrated by the middle eastern people, who are already acquainted with the implications of the MERS epidemic and the contribution of vaccination in combating virus-borne influenza [32]. Previous studies among the Bangladeshi population illustrated a general insufficiency of knowledge regarding the symptoms, severity, and relevant safety measures regarding COVID-19 [33,34]. Such inadequacy may have translated into poor knowledge regarding the vaccine as well. Furthermore, the initiative of a concerted educational program addressing the necessity and efficacy of the COVID-19 vaccine should have been undertaken by concerned authorities, which is still required in order to improve the knowledge level of the general populace. Although certain advertisements have been conducted nationwide regarding the government-funded mass vaccination project, such initiatives also failed to reach the maximum audience [35,36]. Accordingly, when asked about noticing such advertisements, over one-fourth of study participants (27%, *n* = 309) responded negatively.

The COVID-19 pandemic has caused a general rise in negative emotions among the mass populace. In addition, drastic changes in socio-economic structure and lifestyle, along with prolonged isolation and a lack of social interactions, have aggravated fear and anxiety among people. Ultimately, such a heightened level of negativity has adversely affected general confidence in vaccines. Moreover, such negativity has also created space for disseminating misinformation, conspiracy theories, and anti-vaccine sentiment among people [37]. Furthermore, influences from social and political platforms have frequently interfered with effective mass communication from public health experts and deteriorated the public’s perception of the vaccine [38].

COVID-19 vaccine acceptance has suffered remarkably because of the surprisingly accelerated development of the vaccines and the absence of the sufficient safety profiles thereof. Multiple concerns arising from all over the world regarding the possible adverse effects of the vaccines, especially thrombosis and thrombocytopenia, have already resulted in the restriction and even stoppage of vaccine administration in many countries [39]. Evidently, a study in Hong Kong demonstrated that a vaccine willingness of 44.2%, as recorded during the first wave of pandemic, decreased during the third wave (34.8%) due to the emergence of safety concerns [40]. Previous cross-sectional studies had also explained that a lack of adequate effectiveness and safety profiles for vaccines would undermine the overall acceptance of vaccines among the Russian and Indonesian populace [16,41]. Moreover, Bangladesh is currently facing a massive shortage of COVID-19 vaccines because of the irregular and inadequate delivery of vaccines by the Serum Institute of India, which is a collaborative vaccine manufacturer of the Oxford-AstraZeneca vaccine. As a result, by the end of April 2021, around 1.34 million recipients of the first dose were at risk of not receiving the second dose in time. In addition, the absence of any existing contracts with other international sources has also limited the possibility of promptly procuring any vaccines [42]. Such concerns over safety as well as the uncertainty surrounding the availability of vaccines have massively affected the overall perception of the mass population towards vaccination. Consequently, only a tiny fraction (around 4.36%) of the total population has registered for the free vaccination that is being provided by the government [11].

The current study endeavored to evaluate the prevalence of vaccine-related knowledge based on the participants’ education level. Although a previous study also attempted a similar evaluation, their categorization of subjects seemed unsuitable, as all of the subjects who were at or above the undergraduate level were included in the same category. In contrast, subjects below the undergraduate level were classified extensively in primary, lower-secondary, secondary, and higher secondary levels [20]. As per our understanding, Bangladeshi students with a secondary or lower level of education would possess a similar understanding of vaccines since such topics are not covered at these levels. On the contrary, undergraduate and graduate students might exhibit a differential understanding of both vaccine and vaccination processes as they acquire and understand different extents of specialized knowledge based on their area of study. Eventually, we intended to evaluate the vaccine-related knowledge in such categories and observed a significantly higher knowledge level in participants with a Master’s degree or higher compared to that of undergraduate or higher secondary students. The research of Gallè et al. (2021) in Italy adopted a similar generalization and further investigated the effect of background educational level on vaccine-related knowledge. As per their reports, the general knowledge regarding COVID-19 vaccines were significantly higher in students majoring in life sciences or having any form of life science courses in their curriculum [43]. Good knowledge regarding vaccines was also more prevalent among men and older people (older than 40 years). Earlier studies also demonstrated similar trends concerning knowledge about both COVID-19 in general as well as its vaccines [15,28,34]. The implication of a higher susceptibility of the older people towards COVID-19 may have culminated in a more heightened awareness of it, which translated into an adequate knowledge level [44]. Evidently, an Italian study predominantly involving elderly people (mean age 76.6 ± 6.5) demonstrated a very high extent of positive attitudes and acceptance (92.7%) towards COVID-19 vaccination [45]. Moreover, the tendency of the older adults to read newspapers and follow cable channels regularly may have further contributed to their superior understanding of COVID-19 vaccines.

Although the majority of the older participants (74.7%) and those (69.8%) with a Master’s degree or higher have demonstrated good knowledge regarding vaccination, they also exhibited comparatively lower percentages (24.7% and 22.3%, respectively) of good perception levels. Moreover, respondents in the age group of 25–40 years demonstrated the lowest level (19.5%) of positive perception while expressing a decent level of superior knowledge (58.6%) regarding COVID-19 vaccination. A better understanding of the vaccines and the insufficient preparedness of the concerned authorities may have raised more safety and availability concerns within these participants as they came to be dominated by negative perspectives. However, smooth and transparent governmental steps, including several trustable diplomatic measures to procure sufficient vaccines for mass vaccination, might help to achieve herd immunity against the COVID-19 outbreak [46,47,48,49].

Moreover, ensuring vaccination on a mass scale may be attained by the government-mandated enforcement of the issuing and requiring of a special “COVID-vaccinated pass” or something similar, in order to allow free access to public places and social gatherings. In this context, an Italian study provided significant insights on the effectiveness of a “Green pass”, which is a document that has been provided to the Italian population upon completion of vaccination or recovery from COVID-19 and that allows them to move freely through the country and beyond [45]. The study postulated that the introduction of such a strategy, accompanied by comprehensive educational campaigns and the establishment of easily accessible information sources, should improve the mass perception and inclination towards getting vaccinated.

## 5. Strength and Limitations

The major strength of this study lies in the fact that the survey was conducted after COVID-19 vaccination had already started in the country. Therefore, the willingness as projected by the current study is based on the people’s actual impression of the vaccination process while it is ongoing. On the other hand, the previous studies [18,20] explored the willingness for vaccination before it had actually happened and were based entirely on people’s imagination and perception. Moreover, with the ongoing vaccine shortage and emerging reports of adverse reactions, a gradual shift in overall perception and willingness regarding vaccination is also noticeable.

The current study was also challenged by few limitations. Firstly, the current study was self-reporting in nature and that may have introduced bias originating from unnecessary self-consciousness or exaggerated responses of the participants. Secondly, the survey questionnaire was internet-based, meaning it was solely accessible to the participants with at least a minimal internet connection and who were physically capable of navigating it. Finally, the pool of responses received from the participants was devoid of any responses from the extremely marginal populations and concurrent patients who lacked direct access to the internet. Thirdly, the study did not investigate the underlying sources of information based on which the knowledge and perception of the participants were actually developed. Ultimately, whether misinformation, conspiracy theories, and social media have affected the overall outcome of this study remains to be explored. Finally, the participants and their responses were primarily based on the consideration of the ChAdOx1 vaccine of Oxford-AstraZeneca as it was the only vaccine available. However, the availability of multiple vaccines from different sources with varying extents of efficacies has the potential to alter the overall KPW of the mass populace.

## 6. Conclusions

The current study has endeavored to present a projection of the current standing of vaccine-related KPW among the Bangladeshi population. The findings of these analyses have been discussed in detail in order to delineate the gaps in the current policies and the obstacles towards their implementation. This, in turn, may assist the concerned authorities in upgrading their policies accordingly. A structured, comprehensive, and well-versed educational campaign is imminently necessary for the development of proper and adequate vaccination knowledge among the people. Moreover, appropriate media options should be explored in order to disseminate the required knowledge among the underprivileged population of the country. Strict regulations should also be enacted in order to minimize the spread of misinformation and conspiracy theories. Immediate and visionary working plans are warranted from the concerned authorities in order to ensure the adequate availability of vaccines in the coming days. Moreover, up-to-date information regarding the adverse reactions and safety profiles of respective vaccines should be updated regularly in consultation with reputed international journals and circulated accordingly among the people, especially the healthcare professionals. The government should encourage and empower the local pharmaceutical industries to invest in vaccine development. In addition, the government should also diplomatically and administratively converse with the vaccine-producing countries and make arrangements to conduct part of their clinical trials in Bangladesh. Furthermore, to improve public adherence to the vaccination campaign, vaccination completion certificates should be issued by the concerned authorities, and the enforcement of such documents in social and public locations should be mandated. Overall, a multitude of efforts from the authorities in every aspect of vaccine development and administration will improve the general perception of the mass populace, whereas robust educational campaigns will contribute to the improvement of knowledge, thereby leading to an improved willingness and diminished hesitancy among them.

## Figures and Tables

**Table 1:** Demographic characteristics, and knowledge and perception scores (mean ± SD) towards COVID-19 vaccine among general population of Bangladesh (N = 1144).

Variables	Categories	N	%	Knowledge		Perception	
				Mean	SD	Mean	SD
Gender	Male	585	51.1	6.62	1.13	5.09	0.96
	Female	559	48.9	6.33	1.12	5.05	0.99
Age (years)	18–24	797	69.7	6.44	1.07	5.15	1.00
	25–40	169	14.8	6.49	1.27	4.76	0.98
	above 40	178	15.6	6.65	1.26	5.03	0.82
Education (level of schooling)	SSC or below	34	3.0	6.29	1.51	4.94	1.10
	HSC	215	18.8	6.45	1.24	5.13	1.01
	Bachelor	756	66.1	6.45	1.11	5.09	0.98
	Master’s or higher	139	12.2	6.71	0.96	4.94	0.85
Current living area	Rural	289	25.3	6.44	1.22	5.09	1.01
	Urban	855	74.7	6.49	1.10	5.07	0.97
Monthly family income (BDT)	below 25,000	379	33.1	6.34	1.22	5.13	1.00
	25,000–50,000	545	47.6	6.53	1.11	5.02	0.95
	above 50,000	220	19.2	6.60	1.01	5.12	0.99

Note: BDT = Bangladeshi Taka, 1 USD (United States dollar) = 85.81 BDT as of 25 November 2021. SSC = Secondary School Certificate, HSC = Higher Secondary Certificate.

**Table 2:** Chi-square (χ^2^) test of demographic and socio-economic characteristics of Bangladeshi population who have good knowledge, good perception, and willingness of getting vaccine.

Variables		Good Knowledge			Good Perception			Having Willingness		
		N	%	*p*-Value	N	%	*p*-Value	N	%	*p*-Value
Overall	Categories	675	59.0	-	364	31.8	-	588	51.4	-
Gender	Male	384	65.6	**<0.001**	191	32.6	0.537	300	51.3	0.936
	Female	291	52.1	-	173	30.9	-	288	51.5	-
Age (years)	18–24	443	55.6	**<0.001**	287	36.0	**<0.001**	401	50.3	**0.055**
	25–40	99	58.6	-	33	19.5	-	101	59.8	-
	above 40	133	74.7	-	44	24.7	-	86	48.3	-
Highest education (level of schooling)	≤SSC	21	61.8	**0.021**	11	32.4	**0.082**	17	50.0	0.980
	HSC	132	61.4	-	73	34.0	-	108	50.2	-
	Bachelor	425	56.2	-	249	32.9	-	391	51.7	-
	Master’s or higher	97	69.8	-	31	22.3	-	72	51.8	-
Current living area	Rural	174	60.2	0.630	102	35.3	0.142	135	46.7	**0.065**
	Urban	501	58.6	-	262	30.6		453	53.0	
Monthly family income (BDT)	below 25,000	208	54.9	0.134	134	35.4	0.193	201	53.0	0.374
	25,000–50,000	334	61.3	-	163	29.9	-	283	51.9	-
	above 50,000	133	60.5	-	67	30.5	-	104	47.3	-
Knowledge	Poor	-	-	-	139	29.6	0.187	199	42.4	**<0.001**
	Good	-	-	-	225	33.3	-	389	57.6	-
Perception	Poor	-	-	-	-	-	-	381	48.8	**0.001**
	Good	-	-	-	-	-	-	207	56.9	-

**Table 3:** Logistic regression analysis of knowledge, perception, and willingness to get COVID-19 vaccine of general population of Bangladesh.

Variables	Categories	Knowledge				Perception				Willingness			
		AOR	*p*-Value	95% CI		AOR	*p*-Value	95% CI		AOR	*p*-Value	95% CI	
Gender	Male vs. Female	1.748	**<0.001**	1.367	2.235	1.128	0.364	0.870	1.463	0.872	0.272	0.683	1.113
Age (years)	18–24 vs. above 40	0.467	**<0.001**	0.306	0.714	1.944	**0.003**	1.256	3.008	1.129	0.544	0.764	1.668
	25–40 vs. above 40	0.477	**0.002**	0.298	0.764	0.783	0.358	0.464	1.320	1.824	**0.008**	1.170	2.843
Highest Education (level of schooling)	≤SSC vs. ≥Master’s	0.799	0.588	0.355	1.799	1.320	0.521	0.565	3.080	1.027	0.946	0.471	2.239
	HSC vs. ≥Master’s	0.936	0.796	0.565	1.550	1.152	0.607	0.672	1.975	1.043	0.861	0.649	1.676
	Bachelor vs. ≥Master’s	0.801	0.350	0.504	1.275	1.043	0.870	0.629	1.730	1.126	0.594	0.727	1.744
Current living area	Rural vs. Urban	1.107	0.483	0.833	1.471	1.161	0.318	0.866	1.556	0.719	**0.021**	0.543	0.951
Family income (BDT)	Below 25,000 vs. >50,000	0.665	**0.025**	0.465	0.951	1.303	0.166	0.896	1.895	1.413	**0.055**	0.993	2.012
	25,000–50,000 vs. >50,000	0.980	0.904	0.704	1.363	0.942	0.739	0.665	1.335	1.273	0.142	0.922	1.759
Knowledge	Poor vs. Good	-	-	-	-	0.789	**0.078**	0.606	1.027	0.514	**<0.001**	0.401	0.658
Perception	Poor vs. Good	-	-	-	-	-	-	-	-	0.710	**0.010**	0.548	0.920
	Constant	2.786	0.000	-	-	0.261	0.000	-	-	1.256	0.399	-	-

## Data Availability

Available data are presented in the manuscript and supplementary files.

## Supplementary Materials

The following are available online at https://www.mdpi.com/article/10.3390/vaccines9121449/s1, Table S1: Questions presented to the participants through Google Form in order to evaluate their knowledge, perception and willingness towards COVID-19 vaccination.

## References

[1] Hossain M.J., Rahman S.M.A. (2020). Repurposing therapeutic agents against SARS-CoV-2 infection: Most promising and neoteric progress. Expert Rev. Anti-Infect. Ther..

[2] Hossain M.J. (2020). Impact of COVID-19 pandemic among health care providers in Bangladesh: A systematic review. Bangladesh J. Infect. Dis..

[3] World Health Organization, WHO Coronavirus (COVID-19) Dashboard. https://covid19.who.int/.

[4] Siordia J.A. (2020). Epidemiology and clinical features of COVID-19: A review of current literature. J. Clin. Virol..

[5] World Health Organization Draft Landscape and Tracker of COVID-19 Candidate Vaccines. https://www.who.int/publications/m/item/draft-landscape-of-covid-19-candidate-vaccines.

[6] Hossain M.J., Kuddus M.R., Rashid M.A., Sultan M.Z. (2021). Understanding and dealing the SARS-CoV-2 infection: An updated concise review. Bangladesh Pharm. J..

[7] Hossain M.J. (2021). Social organizations and mass media in COVID-19 battle: A bidirectional approach in Bangladesh. Asia Pac. J. Public Health.

[8] World Health Organization WHO Coronavirus (COVID-19) Dashboard. https://covid19.who.int/region/searo/country/bd.

[9] Anadolu Post, Asia-Pacific, Latest on Coronavirus Outbreak. https://www.aa.com.tr/en/asia-pacific/bangladesh-approves-oxford-astrazeneca-covid-19-vaccine/2099147.

[10] Voysey M., Clemens S.A.C., Madhi S.A., Weckx L.Y., Folegatti P.M., Aley P.K., Angus B., Baillie V.L., Barnabas S.L., Bhorat Q.E. (2021). Safety and efficacy of the ChAdOx1 nCoV-19 vaccine (AZD1222) against SARS-CoV-2: An interim analysis of four randomised controlled trials in Brazil, South Africa, and the UK. Lancet.

[11] Directorate General of Health Services, Government of the People’s Republic of Bangladesh. https://dghs.gov.bd/images/docs/vpr/Covid-19-Vaccination-Update-24-5-2021.pdf.

[12] Lazarus J.V., Ratzan S.C., Palayew A., Gostin L.O., Larson H.J., Rabin K., Kimball S., El-Mohandes A. (2021). A global survey of potential acceptance of a COVID-19 vaccine. Nat. Med..

[13] Lin Y., Hu Z., Zhao Q., Alias H., Danaee M., Wong L.P. (2020). Understanding COVID-19 vaccine demand and hesitancy: A nationwide online survey in China. PLoS Negl. Trop. Dis..

[14] Kourlaba G., Kourkouni E., Maistreli S., Tsopela C.G., Molocha N.M., Triantafyllou C., Koniordou M., Kopsidas I., Chorianopoulou E., Maroudi-Manta S. (2021). Willingness of Greek general population to get a COVID-19 vaccine. Glob. Health Res. Policy.

[15] Al-Mohaithef M., Padhi B.K. (2020). Determinants of COVID-19 vaccine acceptance in Saudi Arabia: A web-based national survey. J. Multidiscip. Healthc..

[16] Harapan H., Wagner A.L., Yufika A., Winardi W., Anwar S., Gan A.K., Setiawan A.M., Rajamoorthy Y., Sofyan H., Mudatsir M. (2020). Acceptance of a COVID-19 vaccine in southeast Asia: A cross-sectional study in Indonesia. Front. Public Health.

[17] Larson H.J., Jarrett C., Eckersberger E., Smith D.M., Paterson P. (2014). Understanding vaccine hesitancy around vaccines and vaccination from a global perspective: A systematic review of published literature, 2007–2012. Vaccine.

[18] Abedin M., Islam M.A., Rahman F.N., Reza H.M., Hossain M.Z., Hossain M.A., Arefin A., Hossain A. (2021). Willingness to vaccinate against COVID-19 among Bangladeshi adults: Understanding the strategies to optimize vaccination coverage. PLoS ONE.

[19] Faasse K., Newby J. (2020). Public perceptions of COVID-19 in Australia: Perceived risk, knowledge, health-protective behaviors, and vaccine intentions. Front. Psychol..

[20] Islam M.S., Siddique A.B., Akter R., Tasnim R., Sujan M.S.H., Ward P.R., Sikder M.T. (2021). Knowledge, attitudes and perceptions towards COVID-19 vaccinations: A cross-sectional community survey in Bangladesh. MedRxiv.

[21] Robertson E., Reeve K.S., Niedzwiedz C.L., Moore J., Blake M., Green M., Katikireddi S.V., Benzeval M.J. (2021). Predictors of COVID-19 vaccine hesitancy in the UK household longitudinal study. Brain Behav. Immun..

[22] Ruiz J.B., Bell R.A. (2021). Predictors of intention to vaccinate against COVID-19: Results of a nationwide survey. Vaccine.

[23] Rzymski P., Zeyland J., Poniedziałek B., Małecka I., Wysocki J. (2021). The Perception and Attitudes toward COVID-19 Vaccines: A Cross-Sectional Study in Poland. Vaccine.

[24] Hossain M.J., Hridoy A., Rahman S.M.A., Ahmmed F. (2021). Major Depressive and Generalized Anxiety Disorders Among University Students During the Second Wave of COVID-19 Outbreak in Bangladesh. Asia Pac. J. Public Health..

[25] COVID-19 Vaccines, World Health Organization. https://www.who.int/emergencies/diseases/novel-coronavirus-2019/covid-19-vaccines.

[26] Larson H.J., De Figueiredo A., Xiahong Z., Schulz W.S., Verger P., Johnston I.G., Cook A.R., Jones N.S. (2016). The state of vaccine confidence 2016: Global insights through a 67-country survey. EBioMedicine.

[27] Saied S.M., Saied E.M., Kabbash I.A., Abdo S.A.E.F. (2021). Vaccine hesitancy: Beliefs and barriers associated with COVID-19 vaccination among Egyptian medical students. J. Med. Virol..

[28] Hossain M.J., Kuddus M.R., Rahman S.A. (2020). Knowledge, Attitudes, and Behavioral Responses Toward COVID-19 During Early Phase in Bangladesh: A Questionnaire-Based Study. Asia Pac. J. Public Health.

[29] Cordina M., Lauri M.A. (2021). Attitudes towards COVID-19 vaccination, vaccine hesitancy and intention to take the vaccine. Pharm. Pract..

[30] Olomofe C.O., Soyemi V.K., Udomah B.F., Owolabi A.O., Ajumuka E.E., Igbokwe C.M., Ashaolu U.O., Adeyemi A.O., Aremu-Kasumu Y.B., Dada O.F. (2021). Predictors of Uptake of a Potential Covid-19 Vaccine Among Nigerian Adults. medRxiv.

[31] Sallam M. (2021). COVID-19 vaccine hesitancy worldwide: A concise systematic review of vaccine acceptance rates. Vaccine.

[32] Al-Qerem W.A., Jarab A.S. (2021). COVID-19 vaccination acceptance and its associated factors among a Middle Eastern population. Front. Public Health.

[33] Hossain M.A., Jahid M.I.K., Hossain K.M.A., Walton L.M., Uddin Z., Haque M.O., Kabir M.F., Arafat S.Y., Sakel M., Faruqui R. (2020). Knowledge, attitudes, and fear of COVID-19 during the Rapid Rise Period in Bangladesh. PLoS ONE.

[34] Paul A., Sikdar D., Hossain M.M., Amin M.R., Deeba F., Mahanta J., Jabed M.A., Islam M.M., Noon S.J., Nath T.K. (2020). Knowledge, attitudes, and practices toward the novel coronavirus among Bangladeshis: Implications for mitigation measures. PLoS ONE.

[35] (2021). Coronavirus Info. https://corona.gov.bd/.

[36] Directorate General of Health Services, Government of the People’s Republic of Bangladesh. https://dghs.gov.bd/index.php/en/component/content/article?id=5393.

[37] Chou W.Y.S., Budenz A. (2020). Considering Emotion in COVID-19 vaccine communication: Addressing vaccine hesitancy and fostering vaccine confidence. Health Commun..

[38] Salmon D., Opel D.J., Dudley M.Z., Brewer J., Breiman R. (2021). Reflections On Governance, Communication, And Equity: Challenges And Opportunities In COVID-19 Vaccination: Article examines the engagement and communication steps necessary to strengthen the COVID-19 vaccine roll out by federal, state, and local governments. Health Aff..

[39] Ledford H. (2021). COVID vaccines and blood clots: Five key questions. Nature.

[40] Wang K., Wong E.L.Y., Ho K.F., Cheung A.W.L., Yau P.S.Y., Dong D., Wong S.Y.S., Yeoh E.K. (2021). Change of willingness to accept COVID-19 vaccine and reasons of vaccine hesitancy of working people at different waves of local epidemic in Hong Kong, China: Repeated cross-sectional surveys. Vaccine.

[41] Tran V.D., Pak T.V., Gribkova E.I., Galkina G.A., Loskutova E.E., Dorofeeva V.V., Dewey R.S., Nguyen K.T. (2021). Determinants of COVID-19 vaccine acceptance in a high infection-rate country: A cross-sectional study in Russia. Pharm. Pract..

[42] Dhaka Tribune. https://www.dhakatribune.com/bangladesh/2021/05/04/oxford-vaccine-to-run-out-in-10-days.

[43] Gallè F., Sabella E.A., Roma P., De Giglio O., Caggiano G., Tafuri S., Da Molin G., Ferracuti S., Montagna M.T., Liguori G. (2021). Knowledge and Acceptance of COVID-19 Vaccination among Undergraduate Students from Central and Southern Italy. Vaccines.

[44] Davies N.G., Klepac P., Liu Y., Prem K., Jit M., Eggo R.M. (2020). Age-dependent effects in the transmission and control of COVID-19 epidemics. Nat. Med..

[45] Gallè F., Sabella E.A., Roma P., Da Molin G., Diella G., Montagna M.T., Ferracuti S., Liguori G., Orsi G.B., Napoli C. (2021). Acceptance of COVID-19 Vaccination in the Elderly: A Cross-Sectional Study in Southern Italy. Vaccines.

[46] Hossain M.J. (2020). Is Bangladesh moving toward herd immunity? Current COVID-19 perspective. Bangladesh J. Infect. Dis..

[47] Hossain M.J., Ahmmed F., Rahman S.M.A., Sanam S., Emran T.B., Mitra S. (2021). Impact of online education on fear of academic delay and psychological distress among university students following one year of the COVID-19 outbreak in Bangladesh. Heliyon.

[48] Bari M.S., Hossain M.J., Akhter S., Emran T.B. (2021). Delta variant and black fungal invasion: A bidirectional assault might worsen the massive second/third stream of COVID-19 outbreak in South-Asia. Ethics, Med. Public Health.

[49] Hossain M.J., Rahman S.M., Emran T.B., Mitra S., Islam M.R., Dhama K. (2021). Recommendation and Roadmap of Mass Vaccination against COVID-19 Pandemic in Bangladesh as a Lower-Middle-Income Country (LMIC). Arch. Razi Inst..

