# Supplementary material for: Knowledge, Perception, and Willingness towards Immunization among Bangladeshi Population during COVID-19 Vaccine Rolling Period
Source: Vaccines (Basel). 2021 Dec 7;9(12):1449. doi: 10.3390/vaccines9121449 (PMC8706921; doi:10.3390/vaccines9121449)
Supplement: Supplementary file 1 [file vaccines-09-01449-s001.zip › vaccines-1427422-supplementary.pdf]

Knowledge, Perception, and Willingness towards Immunization among Bangladeshi Population during COVID-19 Vaccine Rolling Period

Md. Sazzadul Bari, Md. Jamal Hossain, Foyez Ahmmed, Md. Mokelsur Rahman Sarker, Labony Khandokar, Aperajita Paul Chaithy, Farina Aziz, Saikat Mitra, Talha Bin Emran, Md. Saiful Islam, Md. Rabiul Islam and Isa Naina Mohamed

Table S1. Questions presented to the participants through Google Form in order to evaluate their knowledge, perception and willingness towards COVID-19 vaccination.

K1: Do you know that COVID-19 vaccine has been approved by World Health Organization (WHO)?	Yes	1083	94.7
	No	61	5.3
K2: Do you know that vaccination against COVID-19 has been started in Bangladesh?	Yes	1130	98.8
	No	14	1.2
K3: Do you know that government is providing free COVID-19 vaccination for the mass population?	Yes	1068	93.4
	No	76	6.6
K4: Have you seen any government-initiated promotions to encourage COVID-19 vaccination?	Yes	835	73.0
	No	309	27.0
K5: Do you know that registration for COVID-19 vaccination can be performed online?	Yes	1072	93.7
	No	72	6.3
K6: Which country is supplying this vaccine to Bangladesh?	Yes	966	84.4
	No	178	15.6
K7: How many doses of Oxford COVID-19 vaccine is required for immunization?	Yes	957	83.7
	No	187	16.3
K8: What is the percentage of efficacy of COVID-19 vaccine in developing immunization?	Yes	299	26.1
	No	845	73.9
P1: Do you think vaccination will help in controlling the COVID-19 situation?	Yes	855	74.7
	No	289	25.3
P2: Do you think that government should be the sole distributor of COVID-19 vaccine?	Yes	258	22.6
	No	886	77.4
P3: Do you think that the government have the capacity to provide free vaccine to the entire population?	Yes	892	78.0
	No	252	22.0
P4: Do you think COVID-19 vaccine can be made available at an affordable and acceptable price by the private sector?	Yes	876	76.6
	No	268	23.4
P5: Do you think that certain population e.g. older citizens and healthcare professionals, should be given priority in administering COVID-19 vaccination?	Yes	1094	95.6
	No	50	4.4
P6: Do you think that the vaccination of general population should be started only after ensuring complete vaccination of priority groups e.g. older citizens and healthcare professionals?	Yes	1113	97.3
	No	31	2.7
P7: Do you think that the country should resume normal life as soon as the vaccination is started?	Yes	716	62.6
	No	428	37.4
W: Do you have willingness to get COVID-19 vaccine?	Yes	588	51.4
	No	556	48.6

*K represents knowledge-based questions; P represents perception-based questions; W represents willingness-related questions.
